# Exploratory Search on Twitter Utilizing User Feedback and Multi-Perspective Microblog Analysis

**DOI:** 10.1371/journal.pone.0078857

**Published:** 2013-11-12

**Authors:** Michal Zilincik, Pavol Navrat, Gabriela Koskova

**Affiliations:** Institute of Informatics and Software Engineering, Slovak University of Technology in Bratislava, Bratislava, Slovak Republic; University of Warwick, United Kingdom

## Abstract

In recent years, besides typical information retrieval, a broader concept of information exploration – *exploratory search* - is emerging into the foreground. In addition, more and more valuable information is presented in microblogs on *social networks*. We propose a new method for supporting the exploratory search on the Twitter social network. The method copes with several challenges, namely brevity of microblogs called tweets, limited number of available ratings and the need to process the recommendations online. In order to tackle the first challenge, the representation of microblogs is enriched by information from referenced links, topic summarization and affect analysis. The small number of available ratings is raised by interpreting implicit feedback trained by *feedback model* during browsing. Recommendations are made by a *preference model* that models user’s preferences over tweets. The evaluation shows promising results even when navigating in the space of brief pieces of information, making recommendations based only on a small number of ratings, and by optimizing the models to process in real time.

## Introduction

In general, exploratory search means searching for information in order to learn about a certain topic. It usually involves an iterative process of exploration and as the user learns more about the topic (or related subtopics), their goal is refined as opposed to direct search for facts where the information goal is well-defined [1].

Exploratory search [2] is typically related to information retrieval and information seeking on the Internet for relevant web pages, articles or books [3], [4]. Currently, since the popularity of social networks is enormous, a lot of valuable content is shared and presented in the form of microblogs. Social networks provide a platform to express opinions, announce news, share interesting facts, communicate or share content such as articles or photographs. The content has heterogeneous nature and is limited to a very small space (usually up to 140 characters), which poses a significant issue for deeper analysis related to information retrieval tasks. To provide the exploratory search in the domain of social networks, it is necessary to enrich the brief textual content to better represent the microblogs.

We further focus on the Twitter social network, where microblogs are known as tweets. Twitter provides metadata for tweets in the form of simple characteristics, which are often used for the analysis. But these metadata do not have to actually be enough to describe the user’s interests or preferences. Therefore, it is necessary to explore further, combine the content with the metadata and use this information for learning the user’s preferences.

We apply the principles of text summarization inspired by an experiment named *TweetMotif* [5] in order to provide the *topic summarization*, i.e. to extract the most common phrases from a set of tweets. Based on occurrences of the extracted phrases, tweets are categorized into topics. We further train the *feedback model* for modeling and interpreting implicit feedback in order not to burden the users by requiring too much explicit feedback. Rated tweets are used to train the *preference model,* which models user’s preferences over tweets. This model is used for recommending interesting tweets to the user.

The rest of the paper is organized as follows. The overview of related work is given in the following section. Third section introduces the proposed method and describes the details of proposed models. The models are evaluated in the fourth section. The fifth section concludes the paper.

### Related Work

To the best of our knowledge, there is no exploratory search tool for Twitter that tries to learn users’ preferences and estimate their intent while allowing them to explore content related to a specified topic. However, there has been extensive research of partial tasks that the proposed exploratory search method incorporates, such as microblog metadata analysis, recommending microblogs for active users of social networks and exploratory search.

Social networks have become a topic of intensive research [6], [7]. When it comes to summarization, work in this area is diverse. There were published successful attempts to summarize tweets by assigning them topic labels such as politics, technology, sports or entertainment [8]. In addition, extensive comparison of existing and proposed algorithms for trending topics summarization has been conducted [9]. This is the case of the common text summarization; tweets for trending topics are known and the task is to show the user a tweet that sums up the given topic in the best way. A different approach is shown in *TweetMotif*; it categorizes tweets into subtopics, which are extracted according to their occurrence [5]. Chosen phrases characterize corresponding tweets because the phrases that do not often occur on Twitter are assigned higher importance. This is also similar to the approach suggested in [10] which focuses on identifying main topics in a text and then summarizing each topic independently. This method was developed for web sites and it makes use of their structuring and larger amount of text, which does not apply to microblogs.

To make the summarization or similar methods more successful, numerous techniques are known that deal with information scarcity in tweets. A study has been conducted where lexical-based enrichment is utilized to increase the amount of features using character n-grams, word n-grams and orthogonal sparse word bigrams [8]. They also describe techniques for overcoming feature scarcity. Link-based external enrichment consists of using words from expanded forms of URLs that are included in tweets. In addition, by part-of-speech tagging and identifying nouns they hope to get better understanding of the topic of the message.

There have been a lot of attempts to evaluate tweet quality and interestingness based on metadata associated with tweets or their authors. In addition to the most straightforward indicator, the retweet count, influence of URL inclusion, mention and hashtag counts along with tweet author’s follower count or listed count on tweet popularity have been researched [11]. New characteristics based on these measures were proposed as well, e.g. *FollowerRank* [12]. Measures describing affect have also been used, namely ANEW which is a dictionary of 1,030 words using valence, arousal and dominance values to describe emotions connected with these words [13]. There is also related work developed especially for Twitter also known as AFINN; all of the 2,476 words in this dictionary are extracted from microblogs and only valence values are used to describe related emotions [14].

To make better recommendations, similarity of tweets from a user’s Twitter timeline to currently analyzed tweets has been measured [15]. On a social network, authors’ influence on topics and authors’ topical interests can be measured easier than on the web. *TwitterRank* algorithm based on *PageRank* quantifies interestingness of content of authors that user follows by utilizing social network structure and topical influence of authors [16]. In other words, it estimates whom the user follows because of interesting content and whom because of different reasons (e.g. social).

Most of the work has been done in the area of metadata (meaning non-textual characteristics) and improving information quality. On the other hand, we identify a certain lack of research regarding exploratory search specialized for Twitter. Moreover, most of the aforementioned methods try to find mainly static rules for tweet interestingness estimation. Other methods are based on the user’s activities on Twitter, which means that the user needs to actively use this social network which leaves out users interested in content of tweets at irregular intervals. We believe that exploratory search needs to respect user’s interests as they change throughout the exploration and focus on user’s current intent. We operate with the premise that interests differ from user to user and also from topic to topic. Predefined rules of what is interesting cannot satisfy everyone every time they look for content. Current research focuses mainly on the quality of results but many successful techniques described here are not usable in real-time conditions, or they proved useful but still serve more as recommenders for active Twitter users than support for exploration of topics not usually read by a user.

An important issue in supporting exploratory search, when a keyword search is not enough, is proposing new user interfaces and interaction models. Many different interactive forms of search, including faceted browsers [17], model-based dynamic presentation [18] or special interaction models [19], are being considered.

In our method we focus on proposing a user interface model to support exploratory search on Twitter. We perform topic summarization in order to categorize tweets into topics. Finally, we present a contribution to estimating implicit ratings and recommending relevant tweets.

## Materials and Methods

Following the conclusion from previous section, we focus on an exploratory search that both allows the user to get a sense of what kind of content is being published on Twitter with regard to a given topic and that also shows recommendations with respect to what catches the user’s interest at the time of exploration. As mentioned earlier, we assume that interests differ from user to user and also from topic to topic, thus recommendations are made based on implicit and explicit ratings provided during the actual session.

To create a system in a reasonable scale, it is necessary to analyze content in real-time. With performance in mind, many decisions need to be made so that acceptable responsiveness is achieved. We focus on practical utilization of the existing methods, their modification and integration into a system that is able to accomplish the defined goals of exploratory search.

### User Interface and Interaction Model

We propose a *user interface model* that allows the user to read tweets and explore content referenced by URLs, while the system is quietly gathering implicit feedback in addition to offering a possibility for the user to provide explicit feedback. If relationships between the chosen tweet characteristics and feedback are discovered, indication of recommended and not recommended topics and tweets are shown to the user.

After the user authenticates the application through Twitter, he can enter a query consisting of one or more keywords. Afterwards, the phase of processing takes place. It is the only phase, which creates significant time delay during interaction with the user. This delay is used for displaying simple instructions for using the application. In an implemented prototype, the processing phase usually takes from 30 seconds to 3 minutes.

Two hundred tweets are fetched via Twitter API based on an entered query. After removing duplicates, there are usually between 120 and 180 tweets left. The data are parsed and saved to the database.

We extend the information describing tweets by features of all URLs that are referenced by them. When this process is complete, tweets are loaded from the database and the *topic summarization* is performed.

After topics are prepared, they are presented to the user. The user can see a list of topic phrases. He can explore topics by clicking on them, which causes relevant tweets to be displayed. The user can read tweets, click on URLs and read the external content as well. The system gathers implicit and explicit feedback from the user and trains *feedback* and *preference models*. The user can then focus on reading the recommended content rather than spending time reading topics and tweets that he does not find interesting.

### Feedback Model

To create a model of user’s preferences and to make recommendations, it is necessary to know the user’s opinions on information he has already read. Since the characteristics of actually searched information may significantly differ from session to session (e.g. when searching for the information about a particular music band, characteristics of interesting tweets differ from those when searching for the place for holiday and even more when learning about a new research area), the profile of what is interesting is based solely on ratings from the current session. Thus there is only a small number of ratings available.

The ideal expression of a user’s opinion is an explicit rating. However, explicit feedback burdens the user so it is better not to force the user to provide it too often. On the other hand, implicit feedback can be gathered more easily than explicit feedback, but it is harder to interpret.

**Figure 1:**
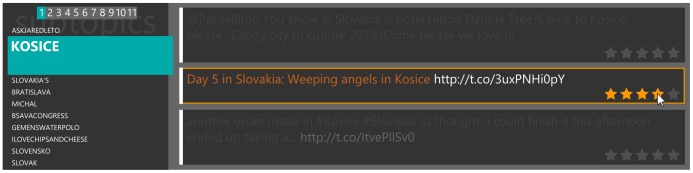
Detail of the user interface. The list of topics can be found on the left side, the topic that the user is reading is highlighted. The rest of the screen shows tweets but only the tweet that is currently hovered by mouse is clearly readable. This helps to increase the accuracy of the attention time measurement because the user is strongly encouraged to move the mouse over the tweet that he is reading at the moment. It can also be seen that the user is choosing four of five stars on the evaluation scale.

As far as we know, there is no published work related to interpretation of the implicit feedback on tweets or similar kind of content. To interpret implicit feedback, it is necessary to learn the difference between the way the user reads interesting and uninteresting tweets. We propose to learn the interpretation of implicit feedback by classifying read but unrated tweets represented by observed values of three attributes: attention time for tweet’s text, click-through for tweets containing URL, and attention time for content referenced by the URLs. A graphical user interface was designed so that it is possible to gather relevant attention time for the tweet’s text. To make the text easily readable, the user needs to move the mouse cursor on the rectangle containing tweet as shown in Figure 1. Text of tweets that are not hovered (that do not have mouse cursor over them) is colored almost exactly like the background. Attention time for content referenced by URL is measured as the time spent between clicking on the link in the tweet and returning to the application. All of these characteristics’ values contain noise, for example the user can click on URL by mistake or can be distracted during reading the tweet or a referenced web page.

A training set consists of tweets that the user read and rated, so explicit feedback is known. We use the commonly used rating scale consisting of five stars. During the processing phase, the user is instructed to rate interesting tweets by four or five stars, uninteresting tweets by one or two stars. Three stars are left to mean neutral attitude. To prove the concept, we chose to categorize tweets into two classes (interesting/uninteresting). Therefore, internally, four and five stars represent interesting tweets and one and two stars represent uninteresting ones.

Since attention times are measured in milliseconds and click-through for a tweet is a binary attribute, we normalize the attribute values using min-max normalization so that values are mapped to an interval <0, 1>.

We chose the Support Vector Machine (SVM) [20] algorithm for creating the *feedback model*. After the user explicitly rates five tweets as interesting and five as uninteresting, first model is trained. As the user rates more tweets, the model is trained repeatedly. To decrease the load of the system, SVM algorithm runs only when five new tweets are rated. The ability of the model to interpret implicit feedback was measured by 10-fold cross-validation, but the score calculation was simplified. Each one of ten validations was considered successful if all the test instances were classified correctly. If some of the tweets from the test set were not classified correctly, we considered the validation to be unsuccessful. Finally, overall score was calculated as a percentage of successful validations from all 10 runs of cross-validation. If the overall score reaches at least 65%, the *feedback model* is used to interpret implicit feedback for read but not rated tweets.

### Preference Model

The *preference model* learns to distinguish which of the unread tweets satisfying user’s query are relevant to the current exploratory search. To do so, topics for the retrieved set of tweets are extracted in the process of *topic summarization* [5]. Then different tweet characteristics are derived and the set of tweets is represented such that in combination of a small number of ratings a classifier can be trained.

#### Topic summarization

First step of the topic summarization process is text preprocessing during which links (URLs) get removed from the tweet text and punctuation and diacritic marks similar to apostrophe are unified, because they are often written incorrectly causing syntactically slightly different phrases for, semantically, the same phrase. Then all characters that are not letters, numbers, apostrophes or whitespaces are removed. Subsequently the text is split into words by whitespaces. This step is simplified and differs in treatment of special characters because we discard Unicode glyphs, emoticons or other strings of punctuation. In this phase, the text is also enriched by words collected from the referenced URLs.

In the second step, n-grams of words (n = 1, 2, 3) are constructed and filtered based on syntax. We remove n-grams that end with words “and”, “of”, “the”, or “a”.

To identify which n-grams represent topics, the extracted phrases are scored by likelihood ratio [5]

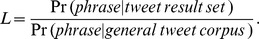



The general tweet corpus consists of tweets that we collected in February 2013 using search queries “the” and “of”. This corpus consists of almost 200,000 tweets. To compensate for n-grams that do not occur in the corpus, probabilities are estimated using *Lidstone smoothing* [5] by
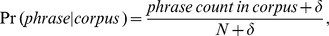
where *N* is the number of all phrases with the specified length *m* in the corpus, *n* is the count of different phrases with the length *m* and *δ* was left with value of 0.5.

The likelihood ratio *L* is also used for filtering general phrases. We remove n-grams for which *L* equals or is less than 10.

The authors of [5] also analyze topic labels with different length and they consider topics unification. For an n-gram and an m-gram where n<m, they check if the n-gram is included in the m-gram, e.g. “flu” and “swine flu”. They propose to ignore the shorter n-gram and leave the m-gram if the tweet set of the longer m-gram is a subset or the same set as the shorter n-gram’s tweet set. Moreover, each pair of tweet sets (with no respect to similarity of the n-grams) is compared. If the Jaccard index for a pair of tweet sets reaches 90%, topics are merged, i.e. one of the topic labels is discarded and the intersection of the two sets of tweets is left. The authors of *TweetMotif* [5] see this as an opportunity to perform further analysis of which topic label should stay and be presented to user. We adopt topics unification with n-grams included in other n-grams and merger of similar topics while random topic label is selected.

#### Using URL for information enrichment

As a result of very limited space for textual content, literally every word in a tweet counts. As suggested, tweets often include an URL [8]. After expanding the shortened URL, more words relevant to the content of web resource can be extracted. However, sometimes URLs do not include any meaningful terms, so we decided to overcome this shortcoming by looking into the page content. We extract the content of HTML title tags extracted from web pages referenced by URLs in tweets and appended it to the tweet text before the topic summarization takes place. This ensures that if several tweets share the link to the same news article, even if they do not contain any common word, they can be grouped by topic phrase(s) extracted from title of linked article.

We also enrich the information describing a tweet by the type of the file the URL references to. The motivation is the following: The proposed method of the Twitter exploratory search is designed with respect to different criteria that the user can have regarding what kind of content is interesting for the current query. For example, the user may be interested in some destination for travelling and the most important tweets for him to read could be those that reference photographs. To support this kind of motivation for exploratory search, we introduce few simple regular expressions used for categorizing URLs into *video*, *audio*, *image* and *other* (unknown) groups. URLs to Instagram, Pinterest, Tumblr, Flickr, Twitter and Facebook photo sharing services are categorized as *image* links, URLs to YouTube and Vimeo are considered to be *video* links, URLs to SoundCloud as *sound* links and the rest falls into the *other* category. Also, we think it would be useful to detect links to research articles, but that would only be possible using a comprehensive page catalog or a quick intelligent content analyzer.

#### Characteristics describing tweets

Generally, we believe that basic metadata affiliated with tweets can support exploratory search as well. Sometimes, a user can be drawn to tweets with URLs because he may want to find resources concerning their area of interest. On the other hand, when looking for overview of people’s opinion of some topic, occurrence of URL may not be important as much as retweet count, author’s listed count or affect.

For describing tweets we observe retweet, URL, mention and hashtag counts. To give the machine learning algorithm a chance to find relationships between these characteristics and the length of a tweet, we also use the number of characters in tweet’s text as an attribute. In addition to URL count we use the estimated content type (image, audio, video and other) as another attribute describing tweets.

For affect observation, we adopted the ANEW [13] approach which uses a list of words with known affective aspect. Emotions are described in a three-dimensional space, where the coordinates consist of values of valence, arousal and dominance. The valence measures how pleasant an emotion is, the arousal describes the intensity and the dominance expresses how controlling the emotion is. The word list contains 1,030 words, for each word the values for these three attributes are defined in a scale from 0 to 10. The values were acquired in a survey. The final representation of the text’s affect is calculated as an average of each attribute’s value for words that match the list. However, many tweets show no affect according to this list of words, so the microblog-optimized AFINN is used as well [14]. AFINN consists of 2,476 words with only one attribute – the value of valence. AFINN matched more tweets than ANEW but still a lot of tweets do not contain any word that can be found in these dictionaries.

In addition to these word lists, we developed regular expressions for extracting almost every commonly used emoticon. Grouping emoticons expressing the same or similar emotions allowed us to estimate valence by mapping nouns or adverbs describing emotions to the words found in ANEW [13] and AFINN [14]. Many tweets contain emoticons while no word is matched against the two dictionaries. That is why we think emoticon extraction can make affective aspect acquisition more successful.

For each tweet we also use three attributes describing the tweet’s author. We observe tweet count, *FollowerRank* [12] and listed count.

Since we adopted topic summarization, we do not use words weighted by TF-IDF or similar weights to represent tweets. That would significantly increase the number of attributes and introduce another delay in the processing phase. We believe that topics based on extracted phrases are sufficient to represent the content of tweets. Topics are defined by the topic phrase and a set of tweets. One tweet can also belong to more than one topic because it can contain more than one phrase. If the user’s interest related to several tweets is expressed by rating, the average rating for the whole topic can be calculated. So if most of the tweets that the user already read for a topic are rated as interesting, the probability that the user will like the unread tweets from the same topic is high. Based on this assumption, we use average rating values for topics that a tweet belongs to and calculate the average from these averages. Since implicit feedback is observed, we use the attention time for tweet’s texts and the click-through ratio as measures instead of explicit rating.

#### Training the preference model

The training set for the *preference model* consists of instances (tweets) that are assigned to one of the groups: interesting or uninteresting. This classification is acquired either directly from the user (rated by 4 or 5 stars for interesting and rated by 1 or 2 stars for uninteresting) or from the *feedback model* for tweets read by the user but not rated. Similarly to the *feedback model*, tweets rated by 3 stars are not assigned to any of the two groups so they are not included in the training set.

The *preference model* is built in the same way as the *feedback model*. Instances are represented by vectors of values for 19 attributes. For affect we use valence, arousal and dominance values by ANEW, valence value by AFINN and valence value based on emoticons. For URLs, their count in the tweet is used; estimated type of web page content is represented by four binary attributes (each one for audio, video, sound and other category). The interestingness of tweets and their authors are represented by hashtag count, mention count, retweet count, FollowerRank, listed count, tweet count and tweet length attributes. To capture the influence of the user’s interest in topics in a tweet, there are average values of the attention times and URL click-through ratios for all topics the tweet belongs to. The values of the attributes are also normalized using the min-max method.

Similarly to the *feedback model*, SVM is used for classification. The model is cross-validated during training and is also used only if the success rate reaches at least 65%. Predictions of used SVM algorithm are output as a real number where −1 represents the uninteresting class and 1 represents the interesting class. Since the model is usually trained only on a few dozens of tweets (e.g. 4), the classifier’s confidence is expected to be low. Therefore, many predictions are represented by values between −0.5 and 0.5. To limit the error of classification, we take all values below −0.1 as assignment to uninteresting class and values above 0.1 as assignments to interesting class. Tweets with values in between are considered as not classifiable by the current model, so no predictions are made for such tweets.

Based on the class predictions for tweets, it is possible to easily calculate a recommendation for topics. If a topic contains at least two unread tweets, the ratio of tweets predicted as interesting out of all predicted tweets for that particular topic is calculated. If the result is higher than 0.6, the topic is flagged as interesting. Otherwise, similar ratio is calculated using number of uninteresting tweets in the numerator. If the result is higher than 0.6, the topic is flagged as uninteresting. If none of the rules apply, neither positive nor negative recommendation is shown.

**Figure 2:**
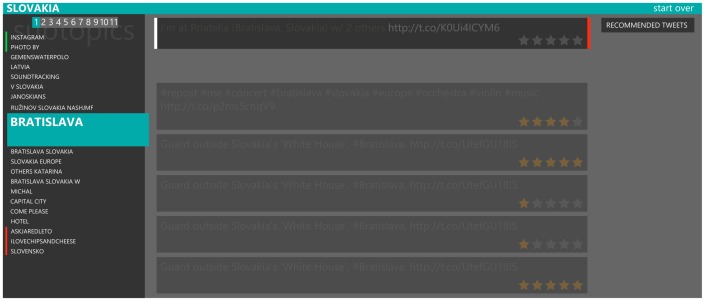
Overview of the user interface with recommendations shown. The recommendations of topics are indicated by color bars to the left of the topic labels. The topics are sorted so that the recommended topics are at the top of the list, the topics without explicit recommendation are next and not recommended topics are last. Even though there are 11 pages of topics, only the first two are recommended, next 15 topics show no prediction of interestingness to the user and the rest of the topics is not recommended. The tweets with translucent background have already been read by the user (and based on the highlighted stars they all have been rated as well). There is also a button in the upper right corner that allows the user to see all recommended tweets from all topics.

In the user interface, the user can see a green bar to the left of the tweet or the topic indicating that the tweet or the topic is predicted to be interesting. For uninteresting tweets and topics a red bar is used. An example is shown in Figure 2. For tweets that were read but not rated by the user, implicit feedback is shown as well. It uses red and green bars on the right side of the tweet.

### Choosing the Machine Learning Algorithm

As mentioned before, we chose SVM [20] as a classifier for both the *feedback* and the *preference models*. Since both models were intended to make binary classifications (interesting/not interesting), for simplicity, we decided to train the same type of the classifier. And the choice was made mainly based on properties of the more complicated problem represented by the *preference model* (operating in 19-attributes space vs. 3-attributes space in the *feedback model*).

The important property of the problem that trains the *preference model* is that for different users, scenarios and exploratory searches in general, different subset of attributes is relevant while others are not important at all. For one search, only tweets referencing to videos are desired. In another exploratory search, topic content can be much more important regardless of the URL type. Thus for training the classifier for a particular session, we might expect the presence of irrelevant attributes. It was shown [21] that SVM performs best for classification, when there are irrelevant attributes. According to the study, it is also one of the most accurate classification algorithms. In addition, SVM can handle dependent attributes, such as valence according to ANEW and AFINN. Attention time to text of tweet might also be dependent on the tweet’s length. And the attention time for referenced content is 0 if the URL click-through ratio is 0, thus there is a strong dependency between these two attributes.

Moreover, SVM can be incrementally trained by new instances. In addition, it is also possible to unlearn a subset of instances [22]. Even though the currently used SVM implementation does not provide the possibility to unlearn instances, application of this feature might speed up the cross-validation procedure.

The last argument for using SVM to classify tweets was its successful application for tweets classification, where several similar attributes were used to represent tweets [11].

We used SVM implementation (http://phpir.com/support-vector-machines-in-php) by Ian Barber with Gaussian RBF kernel. In all runs of SVM, the upper bound for Lagrange multipliers was set to 100. For the RBF kernel, γ of 0.5 was used.

## Experimental Evaluation

To evaluate the proposed method for exploratory search support by making recommendations, we focused on evaluating the *prediction model* and the *feedback model*.

### Evaluating Procedure

We implemented the method as described above and created an experimental version of a system that supports the exploratory search on Twitter. To evaluate the quality of recommendations, they are not presented during the evaluation phase. Instead, the set of tweets predicted as interesting and those predicted as uninteresting by the *preference model* were mixed and presented to the user who was asked to evaluate them.

To correctly evaluate the method, models were trained just after 40 tweets were read by the user. Trained models were used to make predictions for all read and unrated tweets (*feedback model*) and unread tweets (*preference model*). In order not to burden the users, only a subset of those tweets was chosen to constitute an evaluation set. During evaluation, the users were asked to rate all of the tweets in the evaluation set using the scale of 5 stars. Since the set of interesting and uninteresting tweets might be unbalanced, we constitute the evaluation set in a way, that there is approximately the same amount of tweets from both classes. The evaluation set for the *feedback model* consisted of tweets read by the user, but not explicitly rated. This set was made up from up to 15 tweets that were predicted as interesting and up to 15 tweets predicted as not interesting. The evaluation set for the *preference model* consisted of unread tweets. Again, up to 15 tweets predicted as interesting and up to 15 tweets predicted as not interesting were chosen. All of these 60 tweets were picked randomly and if there were less than 15 tweets in some of these four groups, maximum available amount of tweets was chosen.

### Evaluated Alternatives

Since there are many possibilities of how to further preprocess the input of machine learning algorithm in order to achieve a higher success rate, we trained several alternative models to compare their results.

The *feedback model* is trained in two ways. Firstly, the original model was trained as described above. Secondly, an alternative model is trained on the modified training set. Each pair of tweets that have similar implicit feedback, but belong to different classes, was removed from the training set. The similarity of two instances was expressed using cosine similarity with the threshold of 0.99999.

For the *preference model* we constituted alternative models based on the combinations of 5 options. By using the options, we tried to estimate the importance of two groups of attributes for tweets representation: *content type attributes* and *topical attributes*. Under the term *topical attributes* we understand two attributes, which are the averages of attention times and the URL click-through ratios of topics of a tweet. The remainder of the defined set of attributes is considered to be *content type attributes*. In addition, we tested usefulness of implicit feedback and the other option was using the oversampling, if classes in a training set are imbalanced. Similarly to the feedback model, we also experimented with removing similar instances belonging to different classes. The alternative models are labeled by 5-character codes indicating whether particular option is set – coded by the specific letter at a specified position of the code, as can be seen in (Table 1). Otherwise, dash character is used.

**Table 1:** Model options coding.

Position in code	Code letter	Option
1	M	Content type attributes (metadata) are used to represent tweets.
2	T	Topical attributes are used to represent tweets.
3	S	Similar instances belonging to different classes are removed.
4	C	Compensation of imbalance (the amount of instances for classes) – such that the instances from the class with less instances in the training set are duplicated. The result is a balanced training set.
5	F	In addition to explicitly rated tweets, also tweets rated by implicit feedback are used for training (if the success rate from cross-validation reached 65%).

Out of all 32 possible option combinations, there are 8 combinations that cannot be used, since they would only apply preprocessing techniques for not-used attributes. That leaves 24 alternatives. To save time, out of those alternatives, we chose 15 as listed in Table 2, so that it is possible to compare effects of combining the two groups of input attributes and the effect of using the *feedback model*. We focused on the evaluation of *feedback model* more than on the evaluation of preprocessing techniques. So the various combinations of preprocessing techniques were evaluated just in the combination with application of the implicit feedback.

**Table 2:** Results of the preference model – summarizing the alternatives combining selection of attributes (rows – first two letters of the code) and options regarding similar instances removal, compensation of imbalance and implicit feedback interpretation (columns – last three letters of the code).

attributes		(–f)	(-cf)	(s-f)	(scf)	(–)	(s–)
topical (-t)	F1 score (U)	56.50	57.51	88.24	–	56.31	–
	F1 score (I)	50.09	50.74	0.00	–	50.37	–
	success rate	53.52	54.37	78.95	–	53.53	–
	balanced accuracy	54.38	55.46	50.00	–	54.57	–
	*Κ*	8.28	10.21	0.00	–	8.60	–
Contenttype (m-)	F1 score (U)	65.13	73.53	71.79	–	61.93	69.32
	F1 score (I)	41.34	37.04	47.68	–	47.72	50.64
	success rate	56.43	62.73	63.34	–	55.94	62.16
	balanced accuracy	54.10	56.59	59.66	–	55.07	60.39
	*Κ*	8.25	14.43	19.48	–	9.91	20.71
all (mt)	F1 score (U)	59.76	59.50	66.53	66.40	60.48	62.70
	F1 score (I)	46.88	46.81	53.67	53.52	47.41	56.06
	success rate	54.21	54.01	61.14	60.99	54.87	59.65
	balanced accuracy	53.61	53.48	61.53	61.32	54.20	62.61
	*Κ*	7.03	6.75	21.43	21.09	8.21	22.23

We also considered voting as another alternative – *overall voting* – voting of given 15 alternatives was used to make predictions. And the last alternative was built as a *top 3 voting* – voting of three alternative models with the best three scores considering success rate for each class independently (not only the overall success rate).

### Evaluation Metrics

Three basic measures were used for the evaluation. *Success rate* is defined as the number of correctly classified instances (tweets) divided by the number of all instances in the evaluation set.

While evaluating the models, we found out, that training sets are imbalanced. For the *preference model*, 39.9% of instances in a test set belong to the *interesting* class. The proportion is similar to the one in a test set of the *feedback model* where 33.7% of instances belong to the *interesting* class. Thus *success rate* (accuracy) itself may favor classifiers that predict the larger of imbalanced classes more often. To better describe the performance of classifiers on imbalanced data, we also use *balanced accuracy* as suggested in [23].
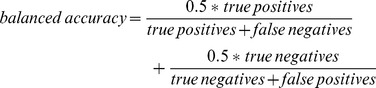



In addition, we measure *κ* (Kappa measure or Cohen’s kappa coefficient).
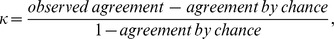

*κ* takes into account the agreement occurring by chance and expresses the success as a proportion of an ideal predictor. These measures are more meaningful when imbalanced sets are used for training, which might be the case if only a small number of ratings for a particular class is available.

To find the most suitable models for *top 3 voting*, we observed how each classifier performs for both classes separately. Precision and recall are calculated for each class and their harmonic mean F1 score is used.

## Results

During the evaluation period, a hyperlink to the online evaluation was posted on Facebook by the author. The hyperlink was also shared by an acquaintance which broadened the group of possible participants. The evaluation was anonymous and users could choose their own query for which tweets were loaded and presented to them. In the end, 25 participants finished their evaluation sessions completely and could be used to compute the results. Data are available at www.fiit.stuba.sk/exploratory_search/data/exploratory_search.zip.

### Evaluating the Feedback Model

The *feedback model* is used only in case of a 65% success rate during cross-validation. Only such models are evaluated, which leaves 12 sessions.

The success rate and *κ* for two alternative models are shown in Table 3. Removing similar instances resulted in a very small training set that affected the results significantly. On the other hand, the original model with all instances predicted the correct class (interesting/uninteresting) for 68% of read but not rated tweets based on attention times for text and content referenced by URLs and URL click-through ratios.

**Table 3:** Success rate and Kappa coefficient for two alternatives of the feedback model.

	successrate	balancedaccuracy	Kappa
original model	68.02	65.13	29.91
without similar instances	45.99	54.59	7.18

### Evaluating the Preference Model

The *preference model* that makes recommendations to the user is fundamental in supporting the exploratory search. We evaluated the *preference model* for all of the 25 finished sessions, even if the model did not reach success rate of 65% in cross-validation during training.

As mentioned before, we observed F1 score for both classes – interesting (I) and uninteresting (U) – in addition to the success rate, balanced accuracy and *κ* (Table 2). The models are arranged according to the coding from Table 1. To choose the best three models for *top 3 voting,* we observed values of *κ.*

**Figure 3:**
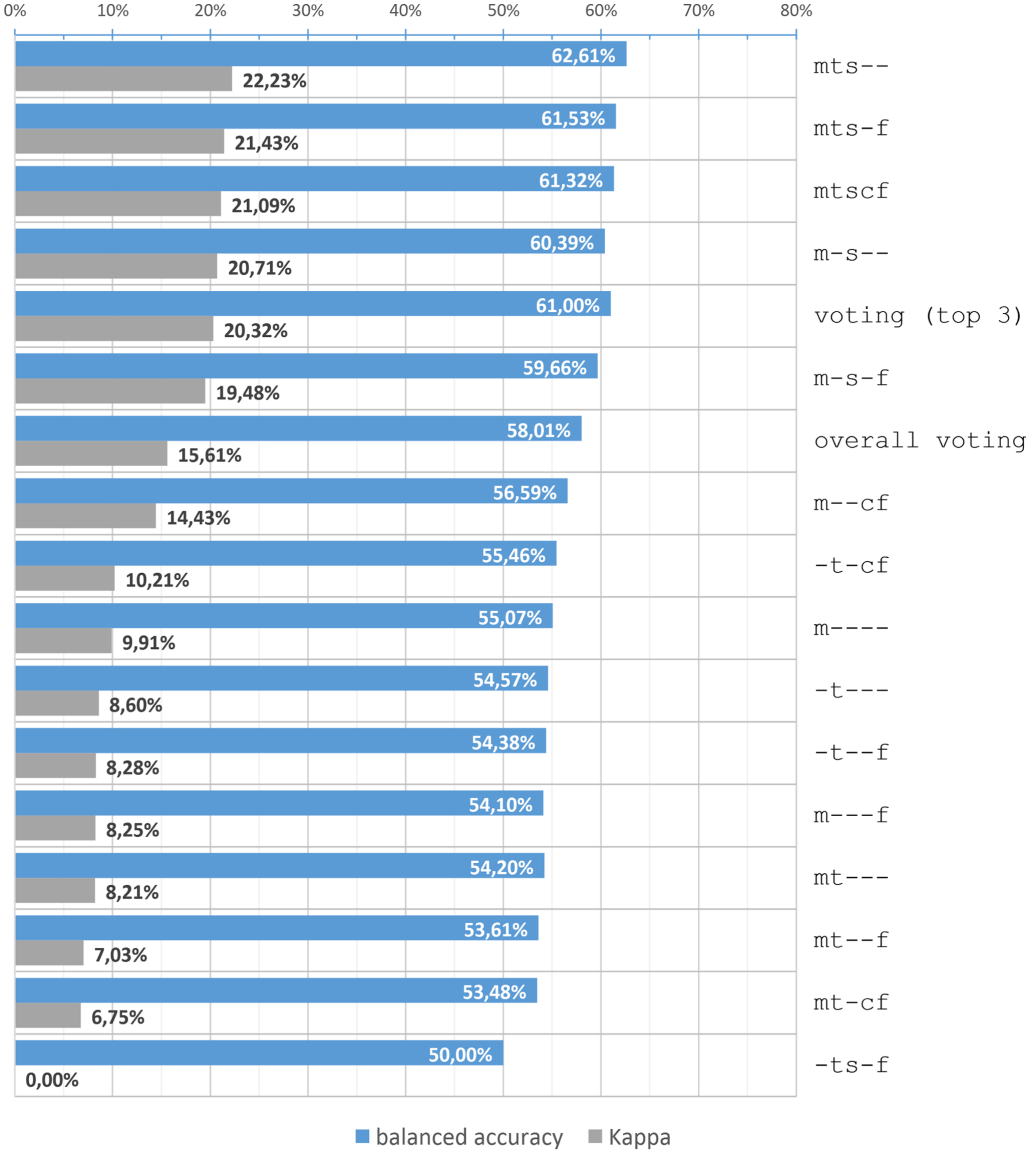
Balanced accuracy and Kappa coefficient for alternatives of preference model. The chart shows the performance of the alternatives of the preference model. The alternatives are sorted by Kappa coefficient. The inputs for top five models were not trained on instances that were similar but belonged to the different classes (–s- in the code), they also used all tweets’ characteristics (m in the code). The model that based its decisions on voting between the top three models (mts–, mts-f and mtscf) did not outperform the m-s– model (trained on tweets’ characteristics with removal of similar instances from different classes).

The final results including *overall voting* and *top 3 voting* are shown in Figure 3. In comparison with a random predictor, the model mts– performed the best. Almost 60% of unread tweets in all sessions were correctly predicted in terms of interestingness to the user based on all 19 attributes with the SVM model trained on only 40 tweets in each session.

Results in the Table 2 are summarized in a way that it is possible to compare the preprocessing methods and the usage of implicit feedback interpretation. When using no preprocessing (besides normalization which is always performed), the difference between using and not using the implicit feedback interpretation is negligible. Unlike we expected, it was slightly counterproductive to use implicit feedback interpretation when all attributes were used. But using the *feedback model* together with either of the preprocessing methods was in most cases better than ignoring unrated tweets.

In case of the –ts-f model, too many similar instances were removed and no predictions could be made, thus rendering this alternative model undecided for 24 of 25 sessions.

## Conclusion

In this paper, we proposed a new method for the exploratory search on Twitter with the aim to support user’s navigation in the content of a diverse environment by giving him recommendations. We concentrated also on allowing the user to focus on the content and explore related topics. Even though the goal is straightforward, to implement practically usable prototype, many considerations had to be made. Appropriate features that are able to describe content from multiple perspectives needed to be chosen, keeping in mind the requirement for a relatively short processing time. Besides the need to make recommendations in real time, there are two other notable challenges. The first one is brevity of tweets. We enriched the content by titles of referenced links, used tweet characteristics provided by Twitter and employed existing methods for affect estimation and topic summarization. The last challenge is the small number of rated tweets in a training set for training the *preference model* that makes recommendations for the user. That is why we also focused on obtaining implicit feedback and proposed the *feedback model*. If successful *feedback model* is built, it can increase the amount of known user’s opinions of content and subsequently enable the *preference model* to make better recommendations.

To the best of our knowledge, there is no similar system that can be compared to our approach, making evaluation by direct comparison impossible. In the experimental evaluation, we showed that it is possible to learn how to interpret implicit feedback even if the observed items are short messages optionally containing URLs. Experimental evaluation also showed that the proposed method reached overall success rate of 68.02%. We believe the evaluation shows, that this kind of approach is appropriate and suitable for further research.

The *preference model* was evaluated in several alternatives and proved useful with only one exception. We showed that the model can provide recommendation to users with a success rate of over 61% based on 19 values describing only 40 tweets that the user read, of which not every tweet is explicitly rated. Even though the success rate may not be high enough to guarantee an immediate success in a commercial application, we conjecture that our proposed method is capable of making a genuine improvement to an exploratory search on Twitter.

In the future, we plan to experiment with learning a long-term *feedback model*, instead of only using the current session to train the *feedback model*. The rationale behind it is that time spent on an interesting tweet may not be topic specific and thus the *feedback model* does not need to be learned for each session from the scratch. A model learned from a larger number of ratings can provide more precise estimates for ratings. More precise rating estimates introduce less noise to a training set of the *preference model,* which might increase accuracy of recommendations.
